# Review of Outcomes of Intramedullary Fibular Nails in the Management of Ankle Fractures in Adults

**DOI:** 10.7759/cureus.75275

**Published:** 2024-12-07

**Authors:** Sanjeevi Bharadwaj, Jibran Ilyas

**Affiliations:** 1 Trauma and Orthopaedics, Barts Health NHS Trust, The Royal London Hospital, London, GBR; 2 Blizard Institute, Queen Mary University of London, London, GBR; 3 Trauma and Orthopaedics, Northwick Park Hospital, London, GBR

**Keywords:** ankle, ankle fractures, distal fibula fracture, fracture, intramedullary fibular nails, intramedullary fixation, intramedullary locked nail, intramedullary nails, lateral malleolus fractures, nails

## Abstract

Distal fibula fractures involving the ankle are one of the most common fractures, often requiring open reduction and internal fixation with plates and screws. The increased incidence of potential wound complications arising from open reduction methods led to a rejuvenated interest in the application of minimally invasive methods like intramedullary nailing of the fibula in the management of ankle fractures and isolated distal fibular fractures. A literature search was performed using Medline, Cochrane, and Embase from 1993 to 2023. Search terms included were fibula OR fibular AND nail OR rod OR intramedullary AND open AND closed for Boolean Operators. In total, 740 studies were found based on our search terms, and 33 abstracts that investigated the functional and clinical outcome after intramedullary fixation of a fibular fracture were reviewed and assessed for eligibility. Nine abstracts were subsequently excluded, as they did not meet inclusion criteria, leaving 24 articles to be included in this review. Although there is inconsistency in outcome reporting, intramedullary fibular nails can be considered a viable treatment option for the management of distal fibula fractures and ankle fractures with good clinical results and a lower rate of complications. However, the scope for the application of intramedullary nailing in the management of ankle fractures in older age diabetic age groups with multiple comorbidities is yet to be determined.

## Introduction and background

Distal fibula fractures involving the ankle are one of the most common skeletal injuries and constitute approximately 9% of all fractures and up to 22.6 % of all lower-limb fractures in the United Kingdom [1,2]. An epidemiological study showed that the majority of the patients sustained isolated distal fibular or lateral malleolus fractures (two-thirds of patients); a quarter of them had bimalleolar fractures, and the rest of the 7% were trimalleolar fractures [1]. Depending on their configuration, certain ankle fractures may necessitate open reduction and fixation to achieve satisfactory reduction, and to achieve this, there are many modalities of ankle fracture fixation. Fracture fixation by open reduction and internal fixation with plates and screws is currently considered the standard modality in the treatment of unstable fractures. However, this open technique increases the possibility of potentially devastating wound complications, leading it to be slowly replaced by minimally invasive surgical management such as intramedullary nailing. The application of nails in the management of fibula fractures was first described in 1972 by Stohrer et al. [3]. An intramedullary nail provides reasonable osseous fixation and stability by allowing minimally invasive percutaneous techniques and providing rotational stability with distal interlocking screws, respectively. This device can be advantageous in achieving early definitive fracture fixation and stabilisation, especially in patients with severe swelling or blisters. Intramedullary devices can also be advantageous in reducing the incidence of possible peroneal tendon irritation by decreasing the need for metalwork removals.

## Review

Materials and method

A literature search was performed systematically using Medline, Cochrane, and Embase for the period 1993 to 2023. Search terms included were fibula OR fibular AND nail OR rod OR intramedullary AND open AND closed for Boolean Operators. Preferred Reporting Items for Systematic Reviews and Meta-Analyses (PRISMA) guidelines were followed throughout the process of the literature search [4]. Articles that evaluated the clinical and functional outcome and the complication rates after intramedullary nailing of fibular fractures in a prospective and/or retrospective cohort.

Our review was restricted to literature involving humans and adult patients above the age of 18 years, with no upper limit set. All the studies included were in the English language and included detailed information on the type of fracture involving or in association with distal fibula fractures, the implant used, the type of surgical treatment that was used, follow-ups, outcomes, and complications. Exclusion criteria included any duplicate results, lack of full access to the original article, studies not involving intramedullary nailing of fibular fractures, biomechanical studies, case reports, review articles, letters to the editor, or comments.

A double-blind review of abstracts was conducted to avoid bias, and the studies that met the eligibility criteria were included. Studies in languages other than English, biomechanical studies, studies restricted to only open or closed injuries, or comparative studies involving non-surgical or conservative treatments were excluded. There is no clear consensus on reporting outcomes. Furthermore, there is no standard outcome measure used between all papers, which makes the data more challenging to interpret. Due to significant heterogeneity in the individual study populations, an accurate meta-analysis could not be performed.

In total, 740 studies were found based on our search terms, and 33 abstracts that investigated the functional and clinical outcomes after intramedullary fixation of a fibular fracture were reviewed and assessed for eligibility. Nine abstracts were subsequently excluded as they did not meet inclusion criteria, leaving 24 articles to be included in this literature review.

Results

In total, 24 studies were selected to review both clinical and functional outcomes [5-27], out of which 6 articles were of level II evidence [10-12,19,22,23], and 19 articles of level III-2 and level III-3 evidence [5-9,13-18,20,21,24-29].

A complete synthesis of data was not possible to perform an accurate meta-analysis because of the heterogeneity of the study population, fixation devices, and the methods of reporting functional outcomes.

Among the 24 studies that involved 988 patients, 5 studies compared nails to plates [10,11,19,22,23]. A total of 115 patients had sustained open ankle fractures and 199 sustained closed ankle fractures with a mean age between 40 and 79 (56.45) and a mean follow-up of 18 months (Range of 4-43 months) across all the studies [5-11,13-16,18-29,29]. The mean union rate was 97.80% across the series, with three patients having nonunion of the distal fibular fracture [7,8,13,15,16,20,21,24,26,28]. One author did not report on unions or non-unions [25]. Most of the nails in our review were in the locked intramedullary nail variety [9,10,13,14,16-24,26-29]. Five studies suggest that the patients were advised 4 to 6 weeks of partial weight bearing or weight bearing as tolerated to the non-weight bearing [8,13,16,25,28]. Six of the authors had not reported their postoperative weight-bearing protocol [7,15,20,21,24,26].

The overall complication rate after fibular nail osteosyntheses ranged from 0% to 12% across the studies [5-11,13-21,23-29]. Five studies compared nails to plates [10,11,19,22,23]. Although the overall complication rates were lower in the fibular nail group when they were compared to the plates with comparable union rates, there was a reported nail-to-plate conversion rate of 9.9 % as shown by Asloum Y et al. in 2014 [10-12,19,23]. Asloum Y et al., in 2014, also reported a high complication rate of 56% with plate osteosynthesis while some authors reported higher complication rates in patients managed with intramedullary fibular fixation [8,11].

The mean union rate across the series in the locked nail cases was 99.1% [9,10,12-23,30-37]. However, the union rate for fibular plate fixation was reported in 5 studies with a mean of 97.6% [10,11,19,23]. Almost all the fractures in the locked series were reported to have united completely except for 112 of the cases where the authors had not reported the union rates [10,33].

Although it was difficult to compare outcomes between studies given the variability of outcome metrics used for the assessment, the functional outcome was mostly reported as good to excellent in those studies in which it was assessed. Functional outcomes were measured using various assessment tools such as Mean American Association of Orthopaedic Surgeons (AAOS) foot & ankle score, Short Form 12 (12SF) physical component score, mean mental component score (MCS), Foot And Ankle Mobility (FAAM) score, Manchester-Oxford Foot Questionnaire (MOX-FQ) summary index, EuroQol Visual Analogue Scale (EQ-VAS) score, EuroQol (EQ)-5D score, RAND questionnaire, Olerud and Molander Ankle Scores (OMS), Baird and Jackson ankle score, Tornetta’s ankle function scoring [38-41].

Lee et al., 2007, reported a series of eight open ankle fractures, which were managed with a Knowles pin. No complications were reported from the Knowles pin group, as opposed to 13.3% of complications in patients treated with a tubular plate. The mean ankle score was 94.2±3.2 points for the Knowles pin group and 91.6±5.1 points for the plate group (p=0.28). Though the differences in the mean ankle score between the two groups were not significant, the Knowles pin group had a lower average operative time of 22 min (43 min plate), a smaller average wound size of 4.2 cm (plate 8.1 cms), and a smaller average hospital stay of 3.4±0.42 (range: 1-7 days) in patients treated with a Knowles pin (plate 5.5±0.72; range: 3-9 days) [7].

Lee and Chen et al., 2009 reported a series of 47 patients with open lateral malleolar (AO type-B2) fractures. Twenty-five open lateral malleolar fractures were operated with a Knowles pin, and the rest of the 22 were operated with a lateral plate. All the ankle fractures showed radiological union within six months (100%). There was no difference in achieving good reduction rates in both cohorts. However, the Knowles pin group was found to have lower complication rates and significantly shorter operation time than the plate. The Knowles pin group had lesser implant-associated symptoms, with only 1 (4%) patient complaining of a symptomatic hardware problem, which was addressed by pin removal. The functional outcome score was also good, with excellent results (92%) of the Knowles pin using [8,42].

Stewart et al., 2013, presented a retrospective study of 23 fibular fractures, of which 9 were open fractures, all of which were treated with distally locked retrograde Ender nail fixation. All these 23 fractures had united, resulting in a union rate of 100%. There were no complications except for one patient who had reported tenderness at the site of screw insertion, which resulted in implant removal. There were no wound complications associated with their fibular nail, nor was there any malignment noted on the radiography during follow-up. One patient requested the removal of the distal interlocking screw for tenderness due to the screw loosening [26].

Challagundla et al., 2018, in a retrospective study involving 15 ankle fractures treated with Acumed intramedullary locked fibular nails (Acumed LC, Hillsboro, Oregon, USA), 4 of which were open ankle fractures. They reported no complications involving fracture reduction in the postoperative period with a 100% union rate. Two patients had to undergo screw removals at six months due to screws being close to the join. They reported a good functional outcome with a Mean Foot and Ankle Ability Measure (FAAM) of 78% [13].

Coifman et al., 2019, conducted a retrospective study of 39 patients operated with Acumed fibular nails, out of which 2 were open fractures of the medial malleoli graded I and III by the Gustillo classification. They reported nail removals in three cases due to irritation and two syndesmotic screw removals. One of the patients had a non-union. Two patients had wound infections, one of which was a deep infection necessitating implant removal and debridement.

However, the author had not specified whether these complications were specific to patients who sustained open fractures [16].

Al-Obaidi et al., 2019 conducted a retrospective cohort study involving 39 patients, 14 of which were open fractures treated with a fibula rod system (FRS; Acumed). One patient had a long-standing infection of the lateral wound and, therefore, had to undergo fibular nail removal at six weeks. The reported overall mean Olerud-Molander Ankle Score (OMAS) was 54 (range 0-85). The functional outcome was worse in the open ankle cohort when compared to the closed ankle fractures cohort, which can be attributed to the presence of articular damage and other associated limb-threatening or life-threatening conditions. The mean score was 30 for open ankle fractures and 60 for closed fractures, signifying worse outcomes for open ankle fractures. The mean AAOS ankle score was 64 (range 20-95), and the mean SF-12 physical component score was 39 (range 19-52) [17].

Karkkola et al., 2020, presented a retrospective study involving 42 patients, out of which 10 were open ankle fractures and were treated with Acumed fibular nails. One patient who sustained an open fracture needed a surgical revision of the wound over the medial malleolus. Their reported functional outcomes of the ankle at two years were similar to those reported in previous studies [9,30,43]. The mean restricted range of movements was reported to be lower (21 degrees) on the injured side than on the uninjured side [28].

Brewer et al., 2021, presented a retrospective cohort study of 20 patients, 12 of whom had open ankle fractures treated with an Acumed FRS. They reported a deep infection in one patient, which necessitated a fusion. They reported mean MOX-FQ and EQ-5D-5L scores of 53.6 and 0.649, respectively, at a median of 40 months post-injury and mean EQ-VAS scores of 70 [20,40,41]. They also observed that there was a decreased range of motion (ROM) on the injured side in comparison to the non-injured side, with a 30-degree (range 0-52) mean arc of motion on the affected side in comparison to 52 degrees (range 23-86) on the affected side [20].

Chen et al., 2021, reported a series of 73 patients, 37 of whom were treated with locking plates and 36 were treated with intramedullary nails. Of the 36 who were treated with IM nails, two patients had open malleolar fractures. Of these, 36 of the 34 patients had good fracture reduction and 2 had fair reduction. A fibula displacement of less than 2 mm was reported in one of these patients, and in the other patient, the fibula had moved back 1-4 mm. There was no poor reduction in both groups. There was no significant difference between the two groups in terms of reduction. The operation time (P ¼ 0.025), intraoperative blood loss (P < 0.001), and fracture healing time (P < 0.001) of the intramedullary nail group were significantly lower than those of the locking plate group. There were no significant differences between the two groups in terms of hospital stay and OMAS [24].

Ahmed et al., 2022, in their retrospective study of 95 adult patients with a mean age of 66 years, had 26.3% open fractures, which were treated with fibula nail fixation using the Acumed fibula nail (Acumed) across 2 major trauma centres (MTCs) and 9 trauma units (TUs). All 26.3% of them had soft tissue loss affecting the lateral malleolus. The complication rate for fibular nails in their series was 28.2%. The rate of infection, metal work, non-union/malunion rate, and implant removal rate were reported as 4.2%, 5.2%, 8.4%, and 2.1 %. The length of the hospital stay was 9.4 days. The fracture took 12.5 weeks to unite [21].

Discussion

Twenty-four studies involving 988 patients in total. Nineteen of these studies had both open and closed fractures, involving a total of 314 patients managed by intramedullary fibular nailing. Five studies compared nails to plates [10,11,19,22,23]. Although definitive conclusions may be elusive considering the limited variability in the available data, the results in these studies were found to be good in terms of healing, quality of fracture reduction, and functional outcomes, with a relatively low rate of complications in general. The five studies that compared nails to plates showed lower complication rates of intramedullary nails than plate fixations. However, there were significant limitations concerning the comparison of limited literature in two of the studies. Significantly higher complication rates of 56% with plate osteosynthesis were reported by Asloum et al., which is significantly higher than the clinical norm in the literature and could be attributed to the author’s experience. They also reported skin necrosis in over 30% of patients and complex regional pain syndrome (CRPS) in over 15% of patients from their plate cohort [11]. Lee et al., who had presented a series of ankle fractures treated with a Knowles pin (unlocked device) for intramedullary fixation, reported high complication rates with intramedullary fixations, although higher complication rates of malunion and possible non-union are expected to occur when unlocked intramedullary nails or pins are used for fracture fixations [8]. However, Wang et al., 2015, in their case series of 23 patients managed with unlocked elastic nail fixation. They reported that there were no wound complications like infections or necrosis. All their patients had achieved primary wound healing. Tornetta’s ankle function scoring was excellent in 17 cases, good in 3 cases, and fair in 1 case, and the good-to-excellent rate was 95.2% [25].

Therefore, reaching a plausible conclusion from the literature on this subject is challenging due to these differences in intramedullary fixation and the low quality of articles. However, although in some studies, there were limitations due to poor follow-up and significantly fewer patients in the nail group as opposed to the nail group, the difference in complication rates (13.3% for fibular nail versus 29.0% for plate fixation) was found to be significant as reported by Peeperkorn et al. [27].

White TO et al. (2022) reported significantly fewer wound infections occurred in the nail group (p ¼ 0.002) with no difference in functional outcome or scar satisfaction in one of the highest quality studies on this subject with a prospective randomized controlled trial of nail versus plate in an elderly population. The rates of metalwork removal, loss of reduction, and malunion were equivocal amongst the nail and plate groups (6 for the plate group versus 5 in the nail group) with a comparably lower cost per patient in the nail group because of the possibly higher rates for revision or second surgery in patients treated with plate fixations [22].

There are significant gaps in the literature concerning the cost benefits to the patients and the cost-effectiveness of these implants. Some of the intramedullary fibular implants can be more expensive than more traditional plates because of the former’s relative novelty in the market. Although the cost of an implant was found to have been evaluated by one of these authors, a surgeon’s assessment of implant costs has often been found to be wildly inaccurate [22,43]. A potential advantage of an intramedullary nail is the reduced need for metalwork removal, thereby avoiding a second surgery and a second hospital admission when compared to plate fixation, neutralising the cost-effectiveness of a nail over a plate in general. Intramedullary nails would be especially beneficial in older diabetic patients with frail skin and significant co-morbidities, where these implants can help us achieve a percutaneous fixation of a fracture with a relatively smaller incision, reducing the chance of tissue disruption and local soft tissue irritation from the surgery with a lower incidence of postoperative pain allowing for early comfortable mobilisation and possibly better overall postoperative outcomes.

## Conclusions

Overall, as mentioned previously, the functional outcome was mostly reported as good to excellent in those studies in which it was assessed though no consistency was observed with regards to the usage of tools for the assessment of functional outcome. Although the scope for the application of intramedullary nailing in the management of the ankle in certain exceptional situations like those beyond open fractures and older age diabetic age groups with multiple comorbidities is yet to be determined, we can conclude with the available data that an intramedullary fibular nail is a viable treatment option for the management of distal fibula fractures and ankle fractures with reasonably good clinical results and a lower rate of complications.

## References

[1] Court-Brown CM, McBirnie J, Wilson G (1998). Adult ankle fractures--an increasing problem?. Acta Orthop Scand.

[2] Kaye JA, Jick H (2004). Epidemiology of lower limb fractures in general practice in the United Kingdom. Inj Prev.

[3] Stöhrer M, Franke D (1972). Nail osteosynthesis of distal extra-articular fractures of the lower leg [Article in German]. Monatsschr Unfallheilkd Versicher Versorg Verkehrsmed.

[4] Page MJ, McKenzie JE, Bossuyt PM (2021). The PRISMA 2020 statement: an updated guideline for reporting systematic reviews. Syst Rev.

[5] Pritchett JW (1993). Rush rods versus plate osteosyntheses for unstable ankle fractures in the elderly. Orthop Rev.

[6] François M, Inal N, Nassar E, Benrahho A, Moujawaz B (1998). The “epiphysa” fibular nail - 45 cases during 30 months. Our experience and results. Eur J Orthop Surg Traumatol.

[7] Lee YS, Huang HL, Lo TY, Huang CR (2007). Lateral fixation of AO type-B2 ankle fractures in the elderly: the Knowles pin versus the plate. Int Orthop.

[8] Lee YS, Chen SW (2009). Lateral fixation of open AO type-B2 ankle fractures: the Knowles pin versus plate. Int Orthop.

[9] Bugler KE, Watson CD, Hardie AR, Appleton P, McQueen MM, Court-Brown CM, White TO (2012). The treatment of unstable fractures of the ankle using the Acumed fibular nail: development of a technique. J Bone Joint Surg Br.

[10] Bugler KE, White TO, Appleton PT, McQueen MM (2013). A prospective, randomised controlled trial of a fibular nail versus standard open reduction and internal fixation for fixation of ankle fractures in elderly patients. Orthop Proc.

[11] Asloum Y, Bedin B, Roger T, Charissoux JL, Arnaud JP, Mabit C (2014). Internal fixation of the fibula in ankle fractures: a prospective, randomized and comparative study: plating versus nailing. Orthop Traumatol Surg Res.

[12] White TO, Bugler KE, Appleton P, Will E, McQueen MM, Court-Brown CM (2016). A prospective randomised controlled trial of the fibular nail versus standard open reduction and internal fixation for fixation of ankle fractures in elderly patients. Bone Joint J.

[13] Challagundla SR, Shewale S, Cree C, Hawkins A (2018). Intramedullary fixation of lateral malleolus using fibula rod system in ankle fractures in the elderly. Foot Ankle Surg.

[14] Tracey J, Vovos TJ, Arora D, Adams S, Parekh SG (2019). The use of modern intramedullary nailing in distal fibula fracture fixation. Foot Ankle Spec.

[15] Al-Obaidi B, Wiik AV, Bhattacharyya R, Mushtaq N, Bhattacharya R (2019). Fibular nails for open and closed ankle fractures: results from a non-designer level I major trauma centre. J Orthop Surg (Hong Kong).

[16] Coifman O, Bariteau JT, Shazar N, Tenenbaum SA (2019). Lateral malleolus closed reduction and internal fixation with intramedullary fibular rod using minimal invasive approach for the treatment of ankle fractures. Foot Ankle Surg.

[17] Boni G, Sanchez GT, Arliani G, Zelle BA, Pires RE, Dos Reis FB (2019). Safety and efficacy of surgical fixation of fibula fractures using an intramedullary nail: a retrospective observational cohort study in 30 patients. Patient Saf Surg.

[18] Dabash S, Eisenstein ED, Potter E, Kusnezov N, Thabet AM, Abdelgawad AA (2019). Unstable ankle fracture fixation using locked fibular intramedullary nail in high-risk patients. J Foot Ankle Surg.

[19] Badenhorst D, Terblanche I, Ferreria N, Burger MC (2020). Intramedullary fixation versus anatomically contoured plating of unstable ankle fractures: a randomized control trial. Int Orthop.

[20] Brewer P, Murray J, Barr L (2022). Fibula nail fixation in ankle fractures with significant soft tissue compromise: a retrospective cohort study. Eur J Orthop Surg Traumatol.

[21] Ahmed M, Barrie A, Kozhikunnath A, Thimmegowda A, Ho S, Kunasingam K, Guryel E (2022). Fibula nail outcomes in soft tissue compromised ankle fractures. Foot Ankle Int.

[22] White TO, Bugler KE, Olsen L, Lundholm LH, Holck K, Madsen BL, Duckworth AD (2022). A prospective, randomized, controlled, two-center, international trial comparing the fibular nail with open reduction and internal fixation for unstable ankle fractures in younger patients. J Orthop Trauma.

[23] Stake IK, Ræder BW, Gregersen MG, Molund M, Wang J, Madsen JE, Husebye EE (2023). Higher complication rate after nail compared with plate fixation of ankle fractures in patients aged 60 years or older: a prospective, randomized controlled trial. Bone Joint J.

[24] Chen H, Li Z, Yang D, Wang P, Niu J, He X, Wu G (2021). Clinical study of intramedullary nailing fixation for the treatment of Danis-Weber B in lateral malleolus fracture. J Int Med Res.

[25] Wang Q, Xu HG, Zhang YC, Dong LJ (2015). Elastic nails for fibular fracture in adult tibiofibular fractures. Int J Clin Exp Med.

[26] Stewart CM, Kiner D, Nowotarski P (2013). Intramedullary nail fixation of fibular fractures associated with tibial shaft and pilon fractures. J Orthop Trauma.

[27] Peeperkorn S, Nijs S, Hoekstra H (2018). Why fibular nailing can be an efficient treatment strategy for AO type 44-B ankle fractures in the elderly. J Foot Ankle Surg.

[28] Karkkola S, Kortekangas T, Pakarinen H, Flinkkilä T, Niinimäki J, Leskelä HV (2020). Fibular nailing for fixation of ankle fractures in patients at high risk of surgical wound infection. Foot Ankle Surg.

[29] Giordano V, Boni G, Godoy-Santos AL, Pires RE, Fukuyama JM, Koch HA, Giannoudis PV (2021). Nailing the fibula: alternative or standard treatment for lateral malleolar fracture fixation? A broken paradigm. Eur J Trauma Emerg Surg.

[30] Kara AN, Esenyel CZ, Sener BT, Merih E (1999). A different approach to the treatment of the lateral malleolar fractures with syndesmosis injury: the ANK nail. J Foot Ankle Surg.

[31] Gehr J, Neber W, Hilsenbeck F, Friedl W (2004). New concepts in the treatment of ankle joint fractures. The IP-XS (XSL) and IP-XXS (XXSL) nail in the treatment of ankle joint fractures. Arch Orthop Trauma Surg.

[32] Rajeev A, Senevirathna S, Radha S, Kashayap NS (2011). Functional outcomes after fibula locking nail for fragility fractures of the ankle. J Foot Ankle Surg.

[33] Kabukcuoglu Y, Kucukkaya M, Eren T, Gorgec M, Kuzgun U (2000). The ANK device: a new approach in the treatment of the fractures of the lateral malleolus associated with the rupture of the syndesmosis. Foot Ankle Int.

[34] Zyskowski M, Wurm M, Greve F (2022). A prospective randomized study comparing functional outcome in distal fibula fractures between conventional AO semitubular plating and minimal invasive intramedullary “photodynamic bone stabilisation”. J Clin Med.

[35] Faber RM, Parry JA, Haidukewych GH, Koval KJ, Langford JL (2021). Complications after fibula intramedullary nail fixation of pilon versus ankle fractures. J Clin Orthop Trauma.

[36] Ramasamy PR, Sherry P (2001). The role of a fibular nail in the management of Weber type B ankle fractures in elderly patients with osteoporotic bone— a preliminary report. Injury.

[37] Tas DB, Smeeing DP, Keizer J, Houwert RM, Emmink BL (2022). Postoperative complications of minimally invasive intramedullary nail fixation versus plate fixation for distal fibular fractures in elderly patients: a retrospective double cohort study in a geriatric trauma unit in the Netherlands. J Foot Ankle Surg.

[38] Olerud C, Molander H (1984). A scoring scale for symptom evaluation after ankle fracture. Arch Orthop Trauma Surg (1978).

[39] The EuroQol Group (1990). EuroQol - a new facility for the measurement of health related quality of life. Health Policy.

[40] Morley D, Jenkinson C, Doll H, Lavis G, Sharp R, Cooke P, Dawson J (2013). The Manchester-Oxford Foot Questionnaire (MOXFQ): Development and validation of a summary index score. Bone Joint Res.

[41] Baird RA, Jackson ST (1987). Fractures of the distal part of the fibula with associated disruption of the deltoid ligament. Treatment without repair of the deltoid ligament. J Bone Joint Surg Am.

[42] Ashman BD, Kong C, Wing KJ, Penner MJ, Bugler KE, White TO, Younger AS (2016). Fluoroscopy-guided reduction and fibular nail fixation to manage unstable ankle fractures in patients with diabetes: a retrospective cohort study. Bone Joint J.

[43] Okike K, O'Toole RV, Pollak AN (2014). Survey finds few orthopedic surgeons know the costs of the devices they implant. Health Aff (Millwood).

